# The Editor's Letter-Box

**Published:** 1921-05-14

**Authors:** H. W. Burleigh

**Affiliations:** Secretary and General Superintendent. Hospital for Epilepsy and Paralysis, Maida Vale, W.


### THE EDITOR'S LETTER-BOX.

### IS A HOSPITAL PAGEANT PRACTICABLE?

*To the Editor of* The Hospital.

Sir,—I would gladly support any movement towards securing greater publicity for the London hospitals, particularly publicity of a combined character.

The London hospitals every day of the year are doing their utmost to call attention to their individual needs, and I have for years urged the adoption of some scheme for placing before the public the reason why the voluntary hospitals and the voluntary hospital principle should be supported. During the war I induced *The Times* to set apart a page at the head of which were printed twelve such reasons, but I had little support from the other hospitals in taking space to give reasons why their particular hospital should be supported.—Yours faithfully,

H. W. Burleigh,
Secretary and General Superintendent.
Hospital for Epilepsy and Paralysis, Maida Vale, W.

